# Background matching can reduce responsiveness of jumping spiders to stimuli in motion

**DOI:** 10.1242/jeb.246092

**Published:** 2024-01-08

**Authors:** Min Tan, Jeremiah Y. O. Chan, Long Yu, Eunice J. Tan, Daiqin Li

**Affiliations:** ^1^ Department of Biological Sciences, National University of Singapore, 14 Science Drive 4, Singapore 117543; ^2^ Centre for Behavioural Ecology & Evolution, College of Life Sciences, Hubei University, Wuhan 430062, Hubei, China; ^3^ Division of Science, Yale-NUS College, 16 College Avenue West, Singapore 138527

**Keywords:** Background matching, Camouflage, Movement, Jumping spiders, Salticidae, Visual acuity, Visual responsiveness

## Abstract

Motion and camouflage were previously considered to be mutually exclusive, as sudden movements can be easily detected. Background matching, for instance, is a well-known, effective camouflage strategy where the colour and pattern of a stationary animal match its surrounding background. However, background matching may lose its efficacy when the animal moves, as the boundaries of the animal become more defined against its background. Recent evidence shows otherwise, as camouflaged objects can be less detectable than uncamouflaged objects even while in motion. Here, we explored whether the detectability of computer-generated stimuli varies with the speed of motion, background (matching and unmatching) and size of stimuli in six species of jumping spiders (Araneae: Salticidae). Our results showed that, in general, the responsiveness of all six salticid species tested decreased with increasing stimulus speed regardless of whether the stimuli were conspicuous or camouflaged. Importantly, salticid responses to camouflaged stimuli were significantly lower compared with those to conspicuous stimuli. There were significant differences in motion detectability across species when the stimuli were conspicuous, suggesting differences in visual acuity in closely related species of jumping spiders. Furthermore, small stimuli elicited significantly lower responses than large stimuli across species and speeds. Our results thus suggest that background matching is effective even when stimuli are in motion, reducing the detectability of moving stimuli.

## Teaser

**Summary:** Jumping spiders display lower levels of response to smaller and faster stimuli against a camouflaged background, suggesting that background matching may be an effective camouflage strategy for moving prey.

## INTRODUCTION

Background matching, one of the most common anti-predator strategies, refers to the similarity between an individual's colour and pattern and its background, thus rendering the individual indistinguishable from its surroundings (Michalis et al., 2017; Ruxton et al., 2019; Stevens and Merilaita, 2011). If the individual is successfully matched to its background, the probability of detection by visually oriented predators can be effectively lowered (Cuthill, 2019; Michalis et al., 2017). However, there may be limitations associated with this strategy. The effectiveness of camouflaging an individual matching a specific background may vary depending on the background (Cuthill, 2019). More importantly, the edges of background-matching individuals could become more noticeable against a patterned background when they move (Hall et al., 2013). This has thus led to the long-held belief that motion and camouflage are mutually exclusive (Cicerone and Hoffman, 1997; Ioannou and Krause, 2009; Regan and Beverley, 1984; Rushton et al., 2007).

Recent studies lend support to the notion that motion does not necessarily ‘break’ camouflage, as there are various adaptations an individual can rely on to prevent detection whilst moving (Brunyé et al., 2019; Hall et al., 2013; Smart et al., 2020; Umeton et al., 2017; Yin et al., 2015). For instance, the presence of distractors or similarly patterned objects can improve the camouflaging effectiveness of an object (Hall et al., 2013). An individual may also restrict movement to short distances, such as the sit-and-pursue tactic observed in spider predators when hunting grasshoppers, which also tend to move shorter distances in response to avoiding predation (Miller et al., 2014). Brunyé et al. (2019) highlighted the importance of speed and pattern contrast in influencing detectability of moving stimuli. Furthermore, fast-moving prey tend to be harder to capture against a heterogeneous background compared with a uniform background (Stevens et al., 2008). A moving animal can also avoid detection by exploiting the receiver's visual constraints through a combination of colour patterns and behavioural adaptations aimed at misleading predators [e.g. protean motion (Chance and Russell, 1959), motion dazzle (Scott-Samuel et al., 2011)] or concealing motion signals [e.g. flicker fusion camouflage (Umeton et al., 2019), motion masquerade (Bian et al., 2016)]. Although there are limitations associated with cryptic strategies in concealing moving individuals, there is some evidence showing that camouflaged targets are harder to detect than uncamouflaged or conspicuous targets (Brunyé et al., 2019; Stevens et al., 2008).

Whether a moving prey can be detected ultimately depends on the visual capabilities of the predator species. Most studies investigating the effect of movement on camouflage have been conducted using humans as predator models (Brunyé et al., 2019; Hall et al., 2013; Murali and Kodandaramaiah, 2018; Stevens et al., 2008). However, studies based on human perception may inevitably differ from those of natural predator–prey systems, thus making it difficult to infer the camouflage efficacy of the moving prey. Furthermore, studies of camouflage and motion are often challenging, as it is difficult to determine the effects of motion on the eyes of potential predators. For instance, prey may be easily detectable to predators with high visual acuity even if the prey moves at high speeds. Many studies of predator–prey interactions focus on the perception of avian predators, which possess excellent vision (e.g. Hodos, 2012; Jones et al., 2007). However, moving prey may be more effectively camouflaged against predators with lower visual acuity, such as insects and spiders (Harland et al., 2012).

With most studies focusing on only one focal predator species (Hämäläinen et al., 2015; Mizutani et al., 2003; Umeton et al., 2019; Watanabe and Yano, 2013), there is limited empirical evidence comparing the effects of movement on prey camouflage across closely related species of natural predators. While closely related species have similar eye morphologies and visual systems, they may differ in terms of visual acuity (Dobberfuhl et al., 2005). For instance, the fruit fly *Drosophila melanogaster* and the killer fly *Coenosia attenuata* have highly similar lenses with similar optical properties, yet *C. attenuata* has much higher levels of spatial acuity and information transfer compared with *D. melanogaster* (Gonzalez-Bellido et al., 2011). To better understand the perception and responses of natural predators, it is thus important to examine a diversity of species, even when closely related.

Jumping spiders (Araneae: Salticidae), the most diverse clade amongst spiders (http://wsc.nmbe.ch, accessed on 3 May 2023), are well known for their high-resolution vision, which plays a critical role in their elaborate courtship and predatory behaviour (Harland et al., 2012; Land, 1985). Many salticid species, such as *Portia* spp. and *Phidippus* spp., have especially high visual acuity relative to their body size (Cerveira et al., 2021; Harland et al., 1999; Land, 1985). Yet, three lineages within the family Salticidae – Lyssomaninae, Spartaeinae and Salticinae – are known to differ greatly in terms of eye design and vision-based predatory strategies (Harland et al., 2012; Maddison, 2015; Maddison and Hedin, 2003; Su et al., 2007). As the structure and function of salticid eyes are relatively well studied (De Agrò et al., 2021; Harland et al., 2012; Jakob et al., 2018; Land, 1985; Zurek et al., 2010), salticids are good models to understand how they respond to signals in motion.

In this study, we investigated the responses of six species of salticids – *Cosmophasis umbratica* Simon, *Menemerus bivittatus* (Dufour), *Phintella vittata* (C. L. Koch), *Portia labiata* (Thorell), *Siler semiglaucus* (Simon) and *Thiania bhamoensis* Thorell *–* to moving stimuli against different backgrounds. First, we determined the responsiveness of salticids to conspicuous, differently sized stimuli in motion (i.e. visual responsiveness assay). Next, we examined the salticid responses to camouflaged stimuli at different speeds (i.e. background-matching assay). We hypothesized that across the species, background matching can effectively camouflage motion, such that the salticid response to background-matching stimuli is lower than the response to conspicuous stimuli. We also hypothesized that salticid responses to motion stimuli depend on species as a result of their potentially varying visual acuity.

## MATERIALS AND METHODS

### Study system and salticid maintenance

**Fig. 1.**
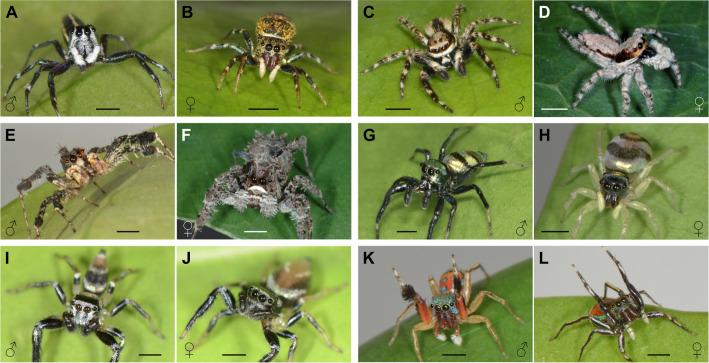
**The six species of salticids used in this study.** *Cosmophasis umbratica* (A) male and (B) female; *Menemerus bivittatus* (C) male and (D) female; *Portia labiata* (E) male and (F) female; *Phintella vittata* (G) male and (H) female; *Thiania bhamoensis* (I) male and (J) female; and *Siler semiglaucus* (K) male and (L) female. Scale bars: ∼2 mm. Image credits: M.T., D.L. and Fan Li.

In this study, we used six salticid species commonly found in Singapore – *C. umbratica* (Salticinae: Saltafresia: Chrysillini), *M. bivittatus* (Salticinae: Saltafresia: Chrysillini), *P. vittata* (Salticinae: Saltafresia: Chrysillini), *P. labiata* (Spartaeinae: Spartaeina), *S. semiglaucus* (Salticinae: Saltafresia: Chrysillini) and *T. bhamoensis* (Salticinae: Saltafresia: Euophryini) (Fig. 1). Additionally, *P. vittata* individuals (*N*=8, 4 females and 4 males) were collected from Ipoh, Malaysia. A total of 191 salticids were used: *C. umbratica* (*N*=39, 20 females and 19 males), *M. bivittatus* (*N*=30, 16 females and 14 males), *P. vittata* (*N*=40, 19 females and 21 males), *P. labiata* (*N*=12, 8 females and 4 males), *S. semiglaucus* (*N*=33, 15 females and 18 males) and *T. bhamoensis* (*N*=37, 19 females and 18 males).

All salticids were housed individually in plastic containers (6×5×5 cm) under controlled environmental conditions (25±1°C, 80±5% relative humidity, 12 h:12 h light:dark cycle). Each salticid was fed with 5–7 laboratory-cultured *D. melanogaster* twice a week and provided with water *ad libitum*. As *P. labiata* prefer spiders as prey (Harland and Jackson, 2000; Jackson and Hallas, 1986; Li et al., 1997) and were larger than the other species tested (M.T., personal observation), they were fed small spiders once every 2–3 weeks. To simulate its natural habitat, we placed a small, dried leaf in the housing container for each individual *P. labiata* for nest building. When the experiments were completed, the salticids were euthanized using carbon dioxide and preserved in 75% ethanol.

### Ethics

This study adheres to the ASAB/ABS guidelines for the use of animals in research, the current legal requirements of Singapore where the research was conducted, and with all National University of Singapore guidelines (OSHM/PI/FOS-289).

### General experimental procedures

**Fig. 2.**
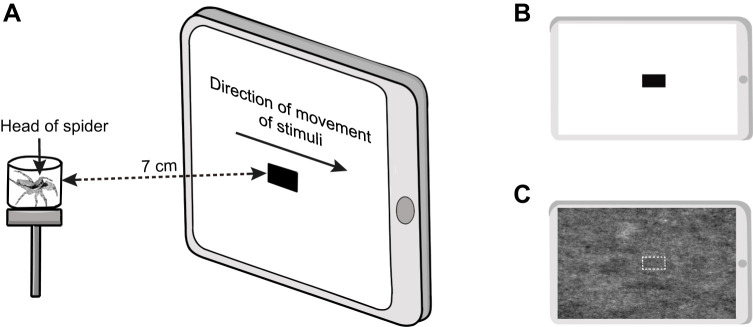
**Experimental setup.** (A) Stimuli were animated to move horizontally across the screen in front of the test salticid in a chamber, which was positioned at the centre of the tablet screen. (B,C) Depiction of the stimuli and backgrounds for the visual responsiveness and background-matching assays, respectively. The white border surrounding the background-matching stimulus is only presented here for clarity and was not displayed in trials.

To determine how the different salticid species respond to the stimuli moving at different speeds in the two assays, we used the experimental method adapted from Umeton et al. (2019), which involved presenting the test subject with computer-generated stimuli moving at different speeds. We carried out two assays – the visual responsiveness assay and background-matching assay – using a similar set-up (Fig. 2A) and procedures except that stimulus type and background were different between two assays (Fig. 2B,C; see details below). To prepare the background and the background-matching stimuli for the background-matching assay, we used images of the bark of rubber trees, *Hevea brasiliensis*. The rubber tree is widely found in Singapore, and several species of salticids (e.g. *M. bivittatus*, *P. labiata*) and their prey are commonly found on the bark of rubber trees (M.T., personal observation). Tree trunks were photographed at breast height (∼1.5 m), at 0.5 m distance using a tripod-mounted Nikon D800 digital SLR camera (Nikon Corp., Tokyo, Japan). The aperture, shutter speed and ISO were f16, 1/60 and 100, respectively. A total of 52 rubber tree trunks were imaged at Pulau Ubin, Singapore. Images were stored in RAW format to avoid loss of information due to compression (Stevens et al., 2007). We used the software ImageJ v.1.49 (Schneider et al., 2012) to calibrate and select a section of the bark photos. To prevent the tree's curvature from distorting the measurement of the texture, only middle sections of the trunk (1200×2400 pixels) were used. We adjusted the brightness and contrast of the images before overlaying and stacking them into one image, thus creating a generic tree bark background. We then converted the background into a monochrome image and cropped the image to fit within the dimensions of a PowerPoint slide [25.4×19.05 cm, 960×720 pixels, 96 pixels per inch (ppi)].

In both the visual responsiveness and background-matching assays, the stimulus comprised a rectangle moving horizontally across the screen. In the visual responsiveness assay, we used a black rectangle stimulus (<1 cd m^−2^; Fig. 2B) presented against the white background. To test for the effects of size on visual responsiveness of motion, we used stimuli of two different sizes – large (63×30 pixels, corresponding to 1.68×0.79 cm, 96 ppi) and small (29×15 pixels, corresponding to 0.76×0.41 cm, 96 ppi). The large and small stimuli are comparable to the body lengths of a salticid and its potential prey, respectively. In the background-matching assay, we used a background-matching stimulus presented against the tree bark background (Fig. 2C). The background-matching stimulus was extracted from the middle section of the background and the dimensions were the same as those used for the large stimulus in the visual responsiveness assay.

Before each trial, we placed the test salticid in a cylindrical viewing chamber positioned on an elevated platform 7 cm from the centre of a tablet screen (iPad 8th generation, Apple Inc.), where the stimulus was displayed (Fig. 2A). Although salticids have been reported to discriminate prey from distances of up to 32 cm, some species, such as *Menemerus* sp., have a maximum discrimination distance of up to 12 cm (Harland et al., 1999). Thus, we deemed 7 cm to be an acceptable distance for our assays. The chamber consisted of a white base (diameter: 1.5 cm), a thin, transparent wall made of clear acetate (thickness: 0.1 cm, height: 0.7 cm) and a transparent lid (adhered to the wall using white tack). As *P. labiata* individuals were generally larger than the rest of the species used, we placed them in a larger viewing chamber (diameter: 3 cm; height: 0.7 cm). The tablet screen was 20.8×15.6 cm, with a pixel resolution of 2160×1620 pixels (264 ppi) and a refresh rate of 60 Hz, which was deemed to be acceptable for this experiment as salticids can perceive flicker up to 40 Hz (Clark and Uetz, 1990). All stimulus animations were made using PowerPoint (Microsoft Corporation), which has a frame rate of 60 frames s^−1^. As salticids are generally diurnal (Foelix, 2011), all trials were conducted during daylight hours (i.e. 09:00–18:00 h). To ensure that the only light came from the iPad, we performed all trials in a dark room in absolute darkness. We recorded the trials using two video cameras (CASIO EX-100, Tokyo, Japan) positioned to record the anterior and posterior views of the test salticid.

The test salticid was first acclimatized in the chamber for 10 min. Next, we waved a thin brush in front of the test salticid to redirect its attention to the screen or gently rotated the chamber using a brush such that the salticid faced the screen. When the salticid turned towards the screen, the stimulus (Fig. 2B,C) was animated to move horizontally across the screen, with the direction of movement of stimuli randomized for each trial. The test ended after the stimulus animation had moved across the screen. The stimuli and background varied depending on the assay.

### Visual responsiveness assay

To determine how salticids respond to an uncamouflaged stimulus, each test salticid was exposed to a black stimulus presented against the white background at different speeds. Each trial consisted of eight presentations, where the stimulus was presented in two sizes – large and small – at four moving speeds [duration of animation on screen: 0.25, 0.5, 1 and 1.5 s, corresponding to speeds: high (83.2 cm s^−1^), medium (41.6 cm s^−1^), low (20.8 cm s^−1^) and very low (13.9 cm s^−1^)]. The moving speeds were decided based on pilot tests, where salticid responses were elicited at these speeds. Furthermore, the speeds were deemed to be ecologically relevant as they are comparable to those of running, jumping or flying arthropods such as beetles (highest running speed: 42 cm s^−1^; Morgan, 1985), hoverflies (highest flight speed indoors: 200 cm s^−1^; Thyselius et al., 2023), salticids (highest jumping speed: 88 cm s^−1^; Brandt et al., 2021), wolf spiders (highest sprint speed: ∼130 cm s^−1^; Nelson and Formanowicz, 2005) and funnel-web spiders (highest sprint speed: approximately 25 cm s^−1^; Pruitt and Husak, 2010), which are potential predators or prey for the tested salticids. The mean luminance of the white screen was approximately 300 cd m^−2^, measured using an illumination meter (Topcon IM-2D, Tokyo, Japan) at the start of each trial. Each presentation was separated by an inter-stimulus black screen interval of at least 50 s, and the stimuli were presented in a random order. During the assay, we observed that, unlike the other species tested, the behaviour of *P. labiata* was affected by the transition from a black to white screen, as *P. labiata* would exhibit freezing behaviour for a long time upon the screen transition. Thus, for *P. labiata*, we used a white screen instead of a black screen during the inter-stimulus interval to minimize potential distractions for this species. Each trial was repeated four times, with at least a 1 day interval between each trial. Thus, a total of 32 stimulus presentations were shown to each salticid – each stimulus type (with two different sizes) presented four times at four speeds.

### Background-matching assay

To determine how salticids respond to a camouflaged stimulus moving at different speeds, salticids were tested using similar procedures to those in the visual responsiveness assay except that only the large background-matching stimuli (Fig. 2C) were presented against the tree bark background in the background-matching assay. Thus, only essential details are described here. To ensure that the salticids were sufficiently responsive to the camouflaged stimulus, only individuals with an average response rate of at least 50% to large black stimuli in the visual responsiveness assay were included in this assay (Fig. S1). *Portia labiata* had very low response rate (only 25% had a response rate of at least 50%) and were thus excluded from this assay. The mean luminance of the screen when the tree bark background was displayed was 45 cd m^−2^, measured using an illumination meter at the start of each trial. Each salticid was exposed to a trial consisting of four stimuli presentations, where the background-matching stimulus was presented at four speeds (corresponding to the speeds tested in the visual responsiveness assay) in a random order. We repeated each experimental trial four times, with at least a 1 day interval between each trial. Thus, a total of 16 stimuli were presented to each salticid.

### Behavioural responses

To determine whether the stimulus was detected by the salticids, we scored the responses of the test salticids following each stimulus presentation (Table 1; Movie 1).

**Table 1.**
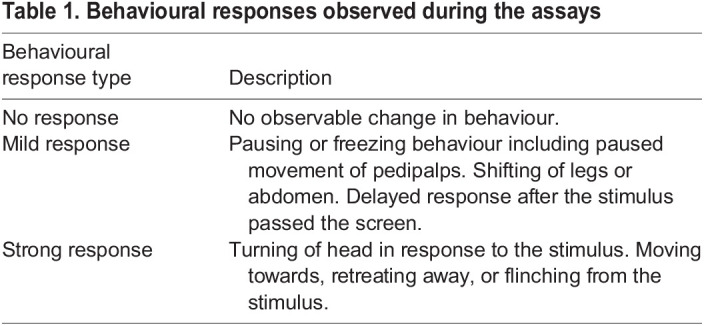
Behavioural responses observed during the assays

### Statistical analysis

We performed two analyses to determine: (i) the effectiveness of background matching for moving stimuli; (ii) whether there are variations in responses among salticid species. For both analyses, we proposed a series of cumulative link mixed models (CLMM) using the ordinal package (https://CRAN.R-project.org/package=ordinal). We coded the behavioural responses of salticids in three levels as the response variable. Salticid identity (ID) and trial number (i.e. sequence of trial for each salticid) were coded as the random intercept and slope, respectively, for all CLMM. We then ranked these models using Akaike's information criterion for small sample sizes (AICc; Burnham and Anderson, 2002) and identified the best-fitting model using model.sel in the MuMIn package (http://R-Forge.R-project.org/projects/mumin). We performed all analyses using R version 4.3.0 (http://www.R-project.org/).

To determine the effectiveness of background matching for moving stimuli, we compared the salticids' responses across both assays and included salticid species, stimulus moving speed, salticid sex and background type as fixed effects. We proposed a total of 14 models, comprising: (i) a null model (1 model); (ii) each fixed effect alone (4 models); (iii) salticid species interacting with the other fixed effects (3 models); (iv) stimulus speed interacting with background/stimulus type (1 model); (v) full models containing all fixed effects with and without interactions (5 models). A comparison of the models can be found in Table S1.

To determine whether there were variations in responses among salticid species, we examined the salticids' responses in the visual responsiveness assay only. We included the same fixed effects and models as above but replaced background type with stimulus size. The proposed models are ranked in Table S2. Additionally, we determined whether there were differences in responses among species at each of the four stimulus speeds tested by using four separate CLMMs to compare the responses among species at each speed in the visual responsiveness assay. For each CLMM, salticid species, stimulus size and sex were included as fixed effects. As the CLMMs only compared the responses of each salticid species to the reference species (i.e. *C. umbratica*), *post hoc* analyses are required to facilitate comparison of species responses. Thus, to determine whether there were significant differences in responses between species, we then conducted pairwise comparison tests for significant effects using the emmeans package (https://CRAN.R-project.org/package=emmeans) to calculate least square means from each CLMM.

## RESULTS

### The effectiveness of background matching

**Fig. 3.**
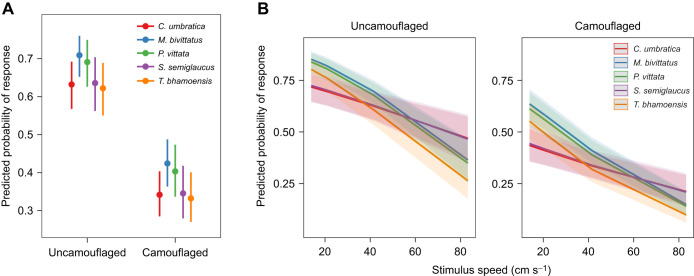
**Predicted probability of salticid responses in the background-matching assay.** Responses to camouflaged and uncamouflaged stimuli (A) and stimulus moving speed (B) across five of the species (*n*=70). The error bars and shaded regions in A and B, respectively, represent the 95% confidence interval (CI).

The model containing species, stimulus moving speed, sex and background type, as well as the interaction between species and stimulus moving speed best predicted the responses of salticids between camouflaged and uncamouflaged stimuli (AICc=3971.4, weight=1; Table S1). Salticids generally showed a significantly higher response to uncamouflaged stimuli when compared with camouflaged stimuli regardless of species (Table 2, Fig. 3), indicating that background matching effectively reduced the detectability of moving stimuli for salticids. Salticids responded less with increasing stimulus moving speed across species, sexes and backgrounds; salticids generally responded differently among species across stimulus moving speeds, sexes and backgrounds. However, females had no significant differences in responsiveness compared with males across species, stimulus moving speeds and backgrounds (Table 2).

**Table 2.**
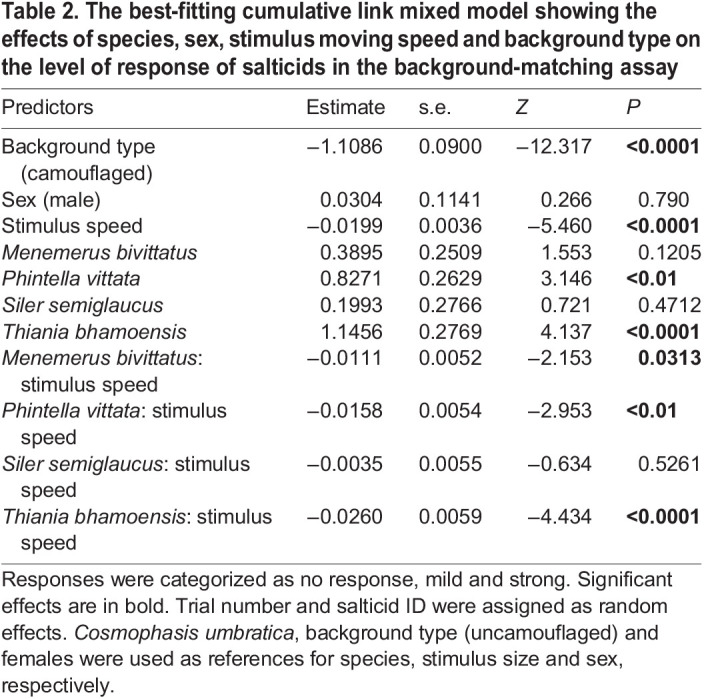
The best-fitting cumulative link mixed model showing the effects of species, sex, stimulus moving speed and background type on the level of response of salticids in the background-matching assay

### Differences in motion detectability among species

**Fig. 4.**
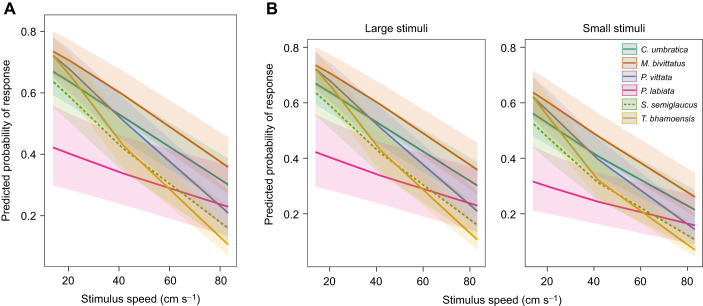
**Predicted probability of salticid responses in the visual responsiveness assay.** Responses across the tested stimulus moving speeds (A) and to large (1.68×0.79 cm) and small (0.76×0.41 cm) stimuli (B) across the six species (*n*=191). Shaded regions represent 95% CI.

In the visual responsiveness assay, where the black stimuli were presented against the white background, the best-fitting model predicting salticid responses included species, stimulus moving speed, stimulus size, sex, as well as the interaction between species and stimulus moving speed (AICc=9872.6, weight=1; Table S2). In general, the conspicuous stimuli elicited different levels of responses among the salticid species tested (Fig. 4A). Salticids responded significantly less with increasing moving speed, and small stimuli elicited significantly lower responses than large stimuli (Table 3 and Fig. 4B). However, sex did not significantly predict salticid responses, though it was included in the best-fitting model (Table 3). Species interacting with stimulus moving speed significantly predicted salticid responses to conspicuous, moving stimuli regardless of size: salticids responded differently to the different stimulus moving speeds depending on species (Table 3).

**Table 3.**
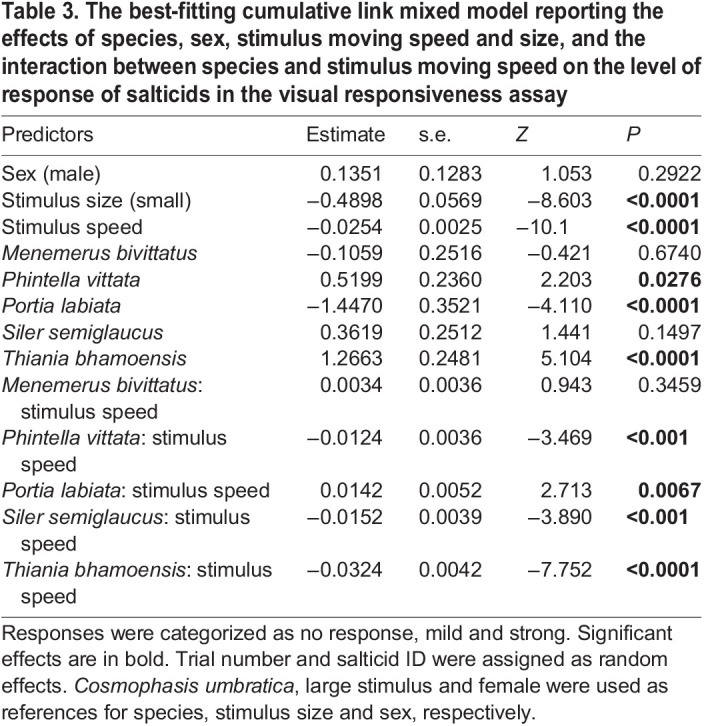
The best-fitting cumulative link mixed model reporting the effects of species, sex, stimulus moving speed and size, and the interaction between species and stimulus moving speed on the level of response of salticids in the visual responsiveness assay

**Fig. 5.**
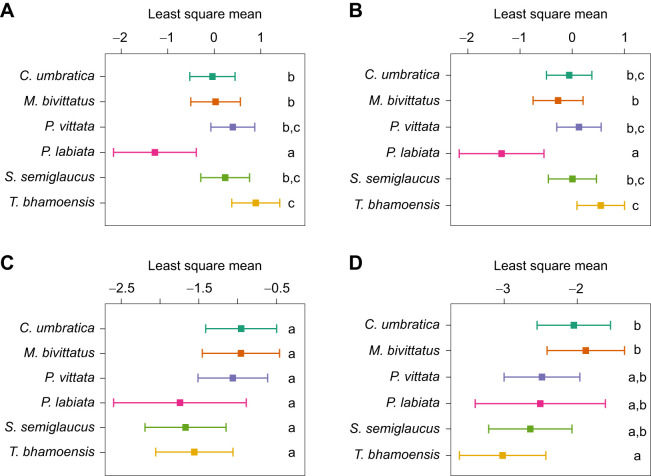
**Least square means for the six salticid species at each speed in the visual responsiveness assay.** Data are for (A) very low (13.9 cm s^−1^), (B) low (20.8 cm s^−1^), (C) medium (41.6 cm s^−1^) and (D) high speed (83.2 cm s^−1^) across the six species (*n*=191). Error bars indicate the 95% CI of the least square means. Different letters indicate Šidák-adjusted comparisons with significant differences.

Pairwise comparisons of the salticids' level of responsiveness across averaged levels of speed, size and sex in the visual responsiveness assay showed that *M. bivittatus*, *P. vittata* and *T. bhamoensis* displayed significantly stronger responses compared with *P. labiata* (Table 4). Furthermore, analyses at specific speeds showed significant differences in the salticids' level of responsiveness (Fig. 4). *Portia labiata* was less responsive and displayed milder responses compared with the rest of the species at most stimulus moving speeds (Fig. 5). Interestingly, *T. bhamoensis* displayed stronger responses compared with the rest of the species at lower speeds (Fig. 5A,B). At the highest speed, *C. umbratica* and *M. bivittatus* displayed significantly stronger responses than *T. bhamoensis* (Fig. 5D). No significant difference in the level of responsiveness was found at the medium speed (Fig. 5C).

**Table 4.**
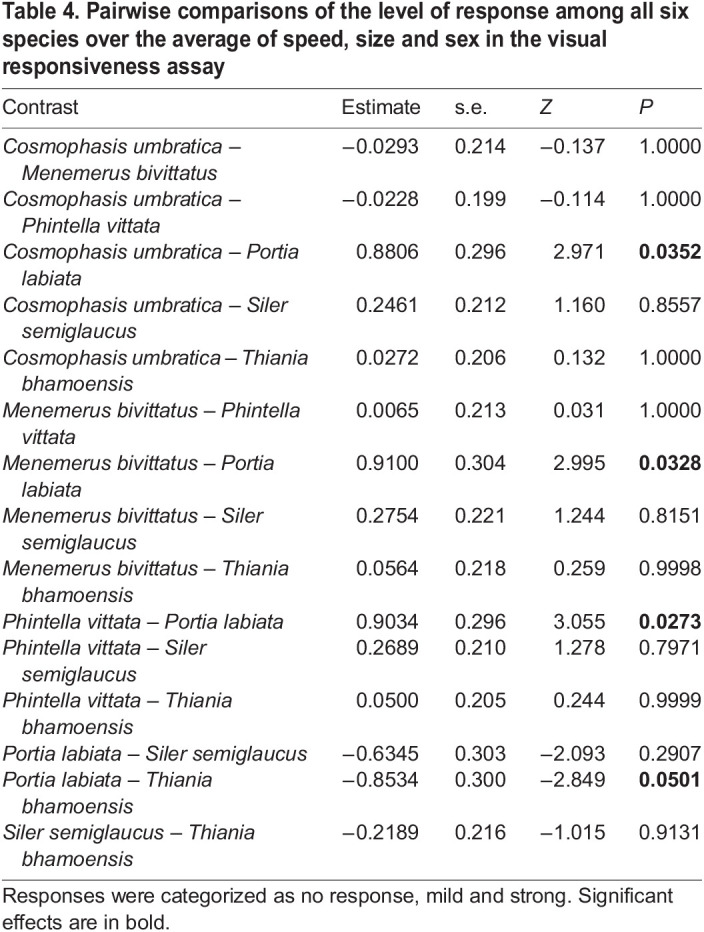
Pairwise comparisons of the level of response among all six species over the average of speed, size and sex in the visual responsiveness assay

## DISCUSSION

Our study demonstrates that salticids displayed lower levels of response to camouflaged than to uncamouflaged stimuli, suggesting that background matching may be an effective camouflage strategy for moving stimuli, especially at higher speeds, in the eyes of salticids. Salticids displayed higher levels of response when the stimulus was larger and moving slower, indicating that the speed of moving stimuli inversely corresponds to detectability. Importantly, we observed significant differences in motion detectability across species when the stimuli were conspicuous, thus suggesting differences in visual acuity and contrast sensitivity across closely related salticid species.

Our findings support our hypothesis that camouflaged moving stimuli, compared with uncamouflaged moving stimuli, were less detectable across the salticid species tested in this study. One possible reason is that the optical performance of the salticid species tested may not be very capable of discriminating stimuli moving against a similarly patterned background. Although little is known regarding the visual acuity of the tested species, our results suggest that the salticids may have difficulty resolving complex backgrounds such as variations in tree bark patterns. Understanding the effectiveness of background matching for camouflage can provide insights into how moving animals exploit the visual constraints of the receiver to escape detection and capture. This is relevant as animals need to move to forage, find mates or escape from potential predators. Previous studies indicate that when a background-matching prey moves, its boundaries become more defined, rendering it more detectable to predators (Hall et al., 2013; Ioannou and Krause, 2009). However, recent empirical studies suggest that prey detectability depends on the background – background-matching prey can be more difficult to detect compared with uncamouflaged prey when coupled with a patterned background (Brunyé et al., 2019; Stevens et al., 2008). Perhaps, prey in motion may be more easily detected if the predator is actively searching for the prey. Unless it is already spotted, prey moving in the predator's peripheral vision can escape detection (Smart et al., 2020). Thus, a well-camouflaged prey would be able to escape detection more easily than a conspicuous one. In addition, the effect of different motion types (e.g. irregular bursts of speed or unpredictable motion trajectory) could further improve the camouflaging effectiveness of background matching in moving prey.

As predicted, the responses of the salticids were negatively correlated with the speed of moving stimuli regardless of species, sex and background type. Our findings are in line with prior studies, which found that prey moving at higher speeds tend to avoid capture (Smart et al., 2020; Stevens et al., 2008). At high speeds, the stimuli could have moved so quickly that it exceeded the motion-processing capabilities of the salticids, thus resulting in lower response levels. Additionally, fast-moving stimuli have an added advantage in avoiding detection if they are moving against a similarly patterned background. Thus, in our study, camouflaged stimuli elicited lower responses compared with conspicuous stimuli, especially at high speeds. Another potential factor may be attributed to salticid attention – although the salticids were looking at the screen during stimulus presentations, their attention may not have been focused specifically on the area of the screen where the stimuli animation occurred. Coupled with factors such as stimulus size, background and even motion type, fast-moving stimuli were less detectable than slow-moving stimuli. Salticids have unique visual systems – the anterior median eyes (AMEs) are involved in the discrimination of colour patterns in stationary objects while the anterior lateral eyes (ALEs) are involved in motion detection (Harland et al., 2012; Jackson and Hallas, 1986). Thus, salticid responses to stimuli moving at high speed may be reactions to movement detection, instead of the appearance of the stimuli. Furthermore, ALEs are also involved in salticid responses to stimuli looming (i.e. rapidly expanding) and receding (i.e. rapidly contracting), which can be important anti-predatory reflexes (Spano et al., 2012). Thus, future studies can consider masking different sets of eyes to better understand the camouflaging effectiveness of differently patterned stimuli, as well as looming or receding stimuli, in motion.

The variation in responses to moving stimuli among salticid species may be explained by their phylogenetic relationship. Five out of the six species tested in this study – *C. umbratica*, *M. bivittatus*, *P. vittata*, *S. semiglaucus* and *T. bhamoensis* – belong to the subfamily Salticinae, whereas *P. labiata* belongs to the subfamily Spartaeinae (Maddison, 2015). Although these five species belong to the same clade, Saltafresia, *T. bhamoensis* belongs to the tribe Euophryini, whereas the other four species belong to the tribe Chrysillini (Maddison, 2015). Species of the subfamily Salticinae are known to have high spatial acuity (Cerveira et al., 2021; Harland et al., 2012; Su et al., 2007), but much remains unknown regarding differences in eye structure, visual acuity and contrast sensitivity between Euophryini and Chrysillini. Additionally, species with high spatial resolving power may face a trade-off in contrast sensitivity (Ryan et al., 2020), which could inevitably influence the detectability of well-camouflaged objects in motion. At low speeds, *T. bhamoensis* displayed stronger responses compared with the rest of the species. Conversely, we observed stronger responses for *C. umbratica* and *M. bivittatus* at high speeds. Our results suggest that Euophryini and Chrysillini salticids show variation in motion responsiveness depending on the speed, with higher visual acuity at low and high speeds, respectively. Unfortunately, there remains limited information regarding the visual acuity of the salticid species tested in this study. To better understand how animals perceive camouflaged stimuli, future studies should consider the visual acuity of the receiver when designing visual stimuli of different sizes, including its field of view, spatial resolving power, light sensitivity and colour perception (Land, 1985).

Variation in responses across species may be the result of adaptations to different habitats (Cerveira et al., 2021; Steinhoff et al., 2020). The salticid species tested in this study were found in habitats with different levels of ambient light. *Cosmophasis umbratica*, *P. vittata*, *S. semiglaucus* and *T. bhamoensis* are typically found in garden bushes and shrubs (Koh et al., 2022). In contrast, *M. bivittatus* and *P. labiata* are more commonly found on tree trunks (Cerveira et al., 2021; Maddison, 2015). *Menemerus bivittatus* can also be found on human infrastructure, such as walls and railings (M.T., personal observation), which can vary greatly in terms of lighting. Luminance is an important factor in motion perception as it is necessary in the discrimination of moving objects (Troscianko et al., 2009). Thus, *M. bivittatus* may be more accustomed to movement in dynamic environmental conditions compared with the other species tested in our study. Variation in luminosity could also have influenced stimulus detectability against different background types. In our study, the textured background had lower mean luminosity compared with the more conspicuous white background. Thus, it is possible that the camouflaged stimuli appear to be less conspicuous than the uncamouflaged stimuli as a result of differences in luminosity. Future research can improve our study by comparing the effectiveness of camouflage on motion using stimuli and background types of similar luminosity or by investigating the detectability of stimuli with varying conspicuousness against one background type.

Low levels of response may not represent low motion-processing capabilities but could be due to species-specific responses. *Portia labiata* displayed low levels of responses compared with the other species of salticids. However, *Portia* species are known to have high spatial acuity and greater resolving power compared with other salticids (Harland et al., 2012; Land, 1985). Spiders of the genus *Portia* behave differently from the majority of salticids, such as adopting a ‘choppy’ and slow gait (i.e. jerking their legs and pedipalps and pausing at irregular intervals) to better camouflage them against their surroundings (Jackson and Blest, 1982), and preferentially practise araneophagy, where they have evolved specialized and complex strategies to capture arachnid prey (Harland and Jackson, 2000). When faced with a potential salticid prey, *Portia* spp. tend to freeze until the prey turn away (Jackson and Blest, 1982). The low level of response of *P. labiata* in our study may therefore be attributed to their prey-stalking strategy, rather than low visual acuity and motion detectability capabilities.

Larger stimuli may elicit greater responses than small stimuli through different mechanisms of perception bias and optimal foraging. When moving, the size of an individual prey can alter the viewer's depth perception. For instance, larger objects may be perceived as being closer to the viewer compared with smaller objects (Scott-Samuel et al., 2011). Furthermore, large targets can appear to move more slowly than small targets (Brown, 1931). Prey body size has been shown to influence a predator's foraging decision, especially as the predator needs to evaluate whether the rewards obtained from prey capture outweigh the costs/risks involved (Juanes, 1992; Nakazawa et al., 2013). Oftentimes, predators must balance potential risks and rewards before pursing prey – a relatively large prey may be energetically costly to predators while a small prey may not yield sufficient energetic returns (Schmitz, 2017). In our study, salticids might have been more responsive to large stimuli as larger stimuli would return greater rewards. At the same time, we acknowledge that the stimuli tested in our study may potentially be regarded as a form of ‘predator’ rather than ‘prey’. As the stimuli used in this study are rectangular and somewhat dissimilar to a natural animal shape, whether the salticids perceive them as a reward or threat remains untested. Given the large stimuli is comparable to the size of a salticid, the salticids may judge the large stimulus to be a greater threat than the small stimulus and, thus, display heightened levels of caution by showing a stronger response.

Although our results indicate that background-matching stimuli are less well detected when in motion, it remains to be shown whether these stimuli evade capture more successfully than non-background-matching stimuli when detected. As our study is one of the first steps in empirically investigating the effectiveness of camouflage in motion, there remains a myriad of questions and factors to be explored. Understanding how motion interacts with camouflage, as well as how animals rely on camouflage to avoid detection when moving, is a crucial next step in this field. To better understand the costs of moving in relation to camouflage, future studies should investigate how camouflaged stimuli are easier to detect when in motion compared with when stationary. In our study, the contrast between the stimuli and background types was quite marked. Future research could examine stimuli with a greater range of conspicuousness and contrast against the background to better understand the types of camouflage pattern that are more effective in avoiding detection when in motion. For instance, Stevens et al. (2011) investigated the effectiveness of differently patterned stimuli in avoiding capture using humans as the study subject. To achieve an ecologically relevant perspective, it is crucial for us to understand the types of patterns that give animals a survival edge over others, as well as the mechanisms through which camouflage strategies work when motion comes into play.

Our findings highlight the importance of stimuli speed and size in camouflaging motion, and how the receiver's responses can vary in motion perception across salticid species within the same subfamily and tribe. We provide empirical evidence on how background matching aids the concealment of moving stimuli. Future studies can further build on these findings to better understand the ecological relevance of camouflage strategies in relation to motion.

## Supplementary Material

10.1242/jexbio.246092_sup1 Supplementary information

## ECR Spotlight

This article has an associated ECR Spotlight interview with Min Tan.
