# Supplementary material for: Background matching can reduce responsiveness of jumping spiders to stimuli in motion
Source: J Exp Biol. 2024 Jan 8;227(1):jeb246092. doi: 10.1242/jeb.246092 (PMC10906486; doi:10.1242/jeb.246092)
Supplement: Supplementary information [file jexbio-227-246092-s1.pdf]

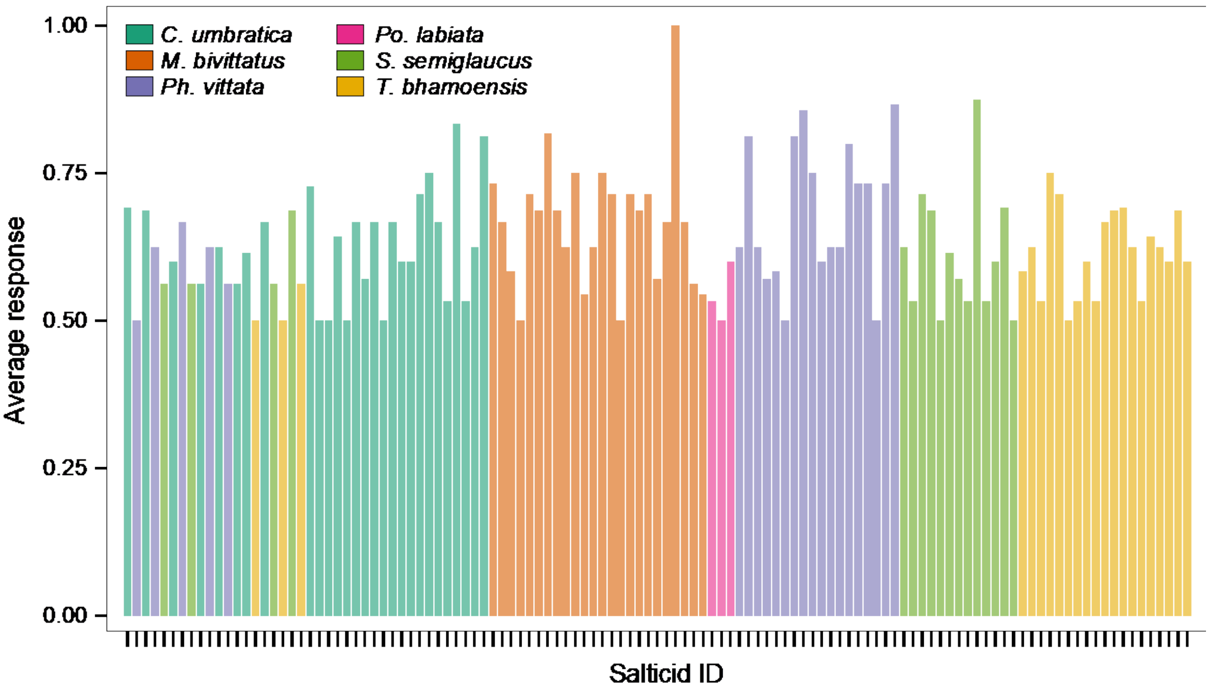

Fig. S1. Salticids with an average response rate to large, black stimuli of at least 50 % in the visual responsiveness assay.

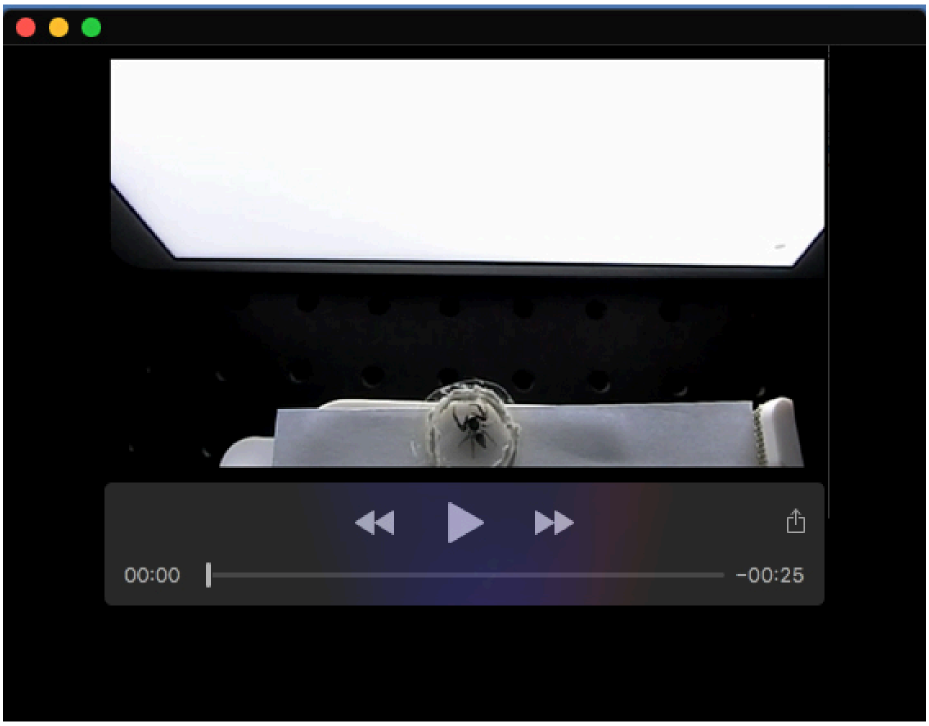

Movie 1. Jumping spider's strong, mild and no response to the stimulus.

Journal of Experimental Biology • Supplementary information

Table S1. Comparison of 14 CLMM to determine the effectiveness of salticid species, sex, stimulus speed and background type on the salticids' response level (i.e. no response, mild response, and strong response)

Predictors	d.f.	logLik	AICc	delta	weight
Background Type + Sex + Stimulus Speed × Species	16	−1969.5	3971.2	0	1
Sex + Stimulus Speed × Background Type + Species	13	−1982.1	3990.4	19.2	0
Stimulus Speed × Background Type	8	−2023.7	4063.5	92.3	0
Sex + Stimulus Speed + Background Type × Species	16	−2022.0	4076.3	105.1	0
Background Type + Sex + Stimulus Speed + Species	12	−2026.2	4076.6	105.4	0
Background Type + Stimulus Speed + Sex × Species	16	−2030.6	4093.4	122.2	0
Species × Stimulus Speed	14	−2058.3	4144.8	173.6	0
Stimulus Speed	6	−2081.4	4174.7	203.5	0
Background Type	6	−2149.1	4310.2	339.0	0
Species × Background Type	14	−2143.2	4314.7	343.5	0
Null	5	−2215.0	4440.1	468.9	0
Sex	6	−2215.0	4442	470.8	0
Species	9	−2213.4	4444.9	473.7	0
Species × Sex	14	−2212.5	4453.2	482.0	0

Table S2. Comparison of 14 CLMM to determine the effectiveness of salticid species, sex, stimulus speed and size on the salticids' response level (i.e. no response, mild response, and strong response)

Predictors	d.f.	logLik	AICc	delta	weight
Sex + Stimulus Size + Stimulus Speed×Species	18	−4916.5	9869.2	0	1
Species × Stimulus Speed	16	−4954.7	9941.5	72.3	0
Sex + Stimulus Size + Stimulus Speed + Species	13	−4978.5	9983	113.8	0
Sex + Stimulus Speed + Stimulus Size × Species	18	−4976.7	9989.6	120.4	0
Stimulus Speed × Stimulus Size	8	−4987.4	9990.7	121.5	0
Sex × Species + Stimulus Speed + Stimulus Size	18	−4984.0	10,004.2	135	0
Sex + Stimulus Size × Stimulus Speed + Species	14	−5070.7	10,169.4	300.2	0
Stimulus Speed	6	−5128.6	10,269.2	400.0	0
Species × Stimulus Size	16	−5453.9	10,939.9	1070.7	0
Stimulus Size	6	−5464.8	10,941.6	1072.4	0
Species	10	−5483.7	10,987.5	1118.3	0
Species × Sex	16	−5481.4	10,995	1125.8	0
Null	5	−5492.9	10,995.9	1126.7	0
Sex	6	−5492.2	10,996.5	1127.3	0

Journal of Experimental Biology • Supplementary information
